# Optimizing outcomes in paraesophageal hernia repair: a novel critical view

**DOI:** 10.1007/s00464-024-11104-2

**Published:** 2024-08-12

**Authors:** Zena Saleh, Vincent Verchio, Yazid K. Ghanem, Johanna Lou, Erin Hundley, Armaun D. Rouhi, Hansa Joshi, Mathew C. Moccia, Dominick M. Scalia, Austin M. Lenart, Zachary A. Ladd, Kenji Minakata, David D. Shersher

**Affiliations:** 1 Department of Surgery, Cooper University Hospital, 3 Cooper Plaza, Suite 411, Camden, NJ USA; 2 Cooper Medical School of Rowan University, Camden, NJ USA; 3 Rowan University, Woodbury, NJ USA; 4 Department of Surgery, Perelman School of Medicine, University of Pennsylvania, Philadelphia, PA USA

**Keywords:** Paraesophageal hernia repair, Critical view, Recurrence, Robotic surgery, Laparoscopic surgery, Surgical mastery

## Abstract

**Background:**

The recurrence rate of paraesophageal hernia repair (PEHR) is high with reported rates of recurrence varying between 25 and 42%. We present a novel approach to PEHR that involves the visualization of a critical view to decrease recurrence rate. Our study aims to investigate the outcomes of PEHR following the implementation of a critical view.

**Methods:**

This is a single-center retrospective study that examines operative outcomes in patients who underwent PEHR with a critical view in comparison to patients who underwent standard repair. The critical view is defined as full dissection of the posterior mediastinum with complete mobilization of the esophagus to the level of the inferior pulmonary vein, visualization of the left crus of the diaphragm as well as the left gastric artery while the distal esophagus is retracted to expose the spleen in the background. Bivariate chi-squared analysis and multivariable logistic and linear regressions were used for statistical analysis.

**Results:**

A total of 297 patients underwent PEHR between 2015 and 2023, including 207 with critical view and 90 with standard repair which represents the historic control. Type III hernias were most common (48%) followed by type I (36%), type IV (13%), and type II (2.0%). Robotic-assisted repair was most common (65%), followed by laparoscopic (22%) and open repair (14%). Fundoplications performed included Dor (59%), Nissen (14%), Belsey (5%), and Toupet (2%). Patients who underwent PEHR with critical view had lower hernia recurrence rates compared to standard (9.7% vs 20%, *P* < .01) and lower reoperation rates (0.5% vs 10%, *P* < .001). There were no differences in postoperative complications on unadjusted bivariate analysis; however, adjusted outcomes revealed a lower odds of postoperative complications in patients with critical view (AOR .13, 95% CI .05–.31, *P* < .001).

**Conclusion:**

We present dissection of a novel critical view during repair of all types of paraesophageal hernia that results in reproducible, consistent, and durable postoperative outcomes, including a significant reduction in recurrence and reoperation.

Paraesophageal hernia refers to the abnormal protrusion of abdominal viscera components through the esophageal hiatus and can be associated with congenital or acquired widening of the diaphragmatic hiatus characterized by weaknesses in the muscular crura [1]. Paraesophageal hernias not only cause reflux symptoms, but various mechanical and obstructive symptoms including dysphagia, vomiting, malnutrition, repeated coughing from aspiration, and pneumonia [2]. Severe complications such as gastric gangrene, volvulus, perforation, and symptoms of cardiac compression may arise, posing life-threatening risks [1–3]. Hiatal hernias can be categorized into four types, which include sliding hiatal hernia with cranial displacement of the lower esophageal sphincter (type 1), true paraesophageal hernias (type II), mixed paraesophageal hernia with a large portion of the stomach in hernia (type III), and massive paraesophageal hernia involving displacement of the stomach and other organs (type IV) (Fig. 1) [4]. Surgical intervention is typically necessary in type II, III, and IV even without symptoms. Symptomatic type I hiatal hernias that are resistant to medical therapy may require surgical intervention [5, 6].

**Fig. 1:**
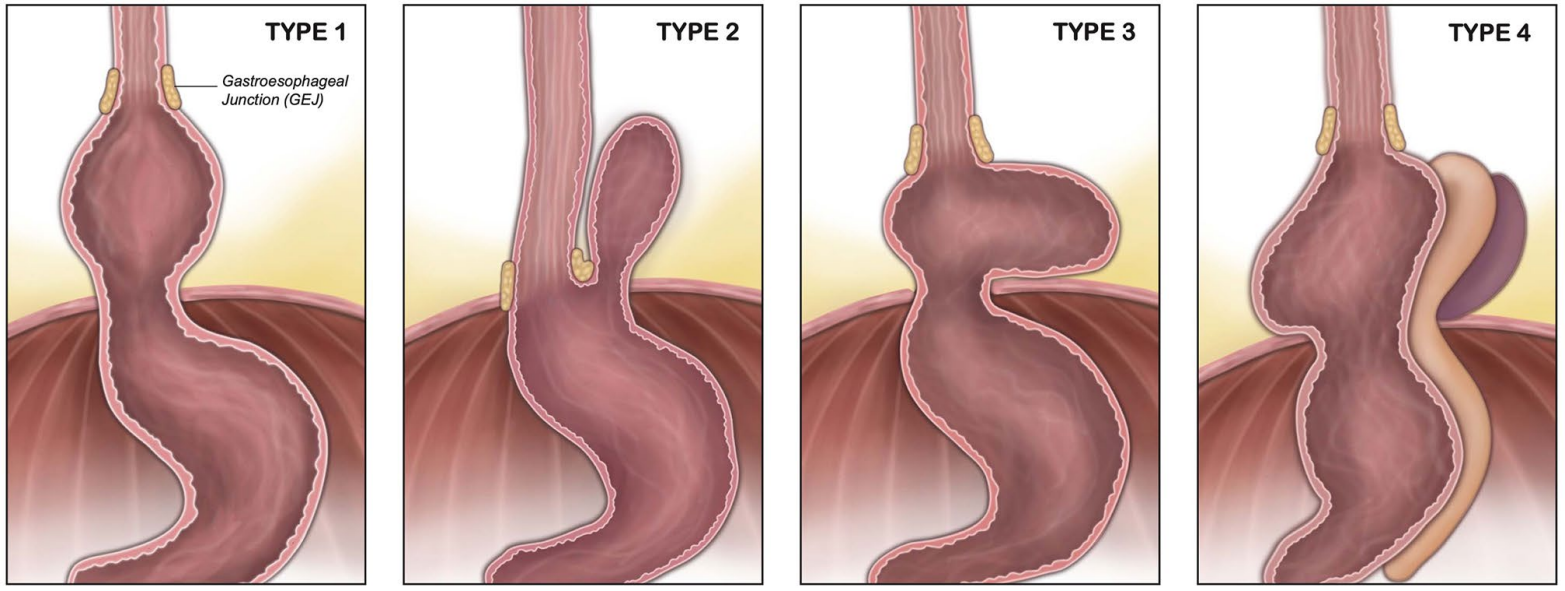
Types of paraesophageal hernia

Principles of standard repair include reduction of the hernia sac, crural closure with or without mesh, addition of a possible lengthening procedure in the setting of a short esophagus to exaggerate length of intraabdominal distal esophagus, and a fundoplication [7]. Robotic and laparoscopic repair is currently the standard treatment for symptomatic paraesophageal hernia with open repair reserved for more complicated and often redo repair [8, 9]. Recurrence following paraesophageal hernia repair (PEHR) is a common complication with rates reported as high as 66% [10]. Factors that may contribute to failure include a shortened intraabdominal esophagus, inadequate crural closure, and obesity with increase in body mass index (BMI) [11–13]. To mitigate the risk of recurrence, the use of mesh to strengthen the crura has been proposed; however, recent findings indicate that regardless of mesh type, the long-term efficacy of mesh and primary suture cruroplasty did not show notable differences [14–16]. Other strategies including the addition of anterior gastropexy and tension free cruroplasty have not been found to influence recurrence rate either [17].

We implemented an aerodigestive program at our institution, which led to the development of a new and reproducible technique that involves utilization of a critical view. We propose that incorporation of a critical view in PEHR can lead to significant improvement in postoperative outcomes and patient satisfaction.

## Materials and methods

A single-center retrospective cohort study was performed that examined operative outcomes in patients who underwent PEHR between 2015 and 2023 with a critical view in comparison to patients who underwent standard repair (IRB 23–107). Patient demographics including age, gender, body mass index (BMI), comorbidities, hernia type, and surgical history were collected. As part of our multidisciplinary aerodigestive program, all patients underwent preoperative evaluation including esophagogastroduodenoscopy (EGD) and computed tomography (CT) for anatomic mapping. Patients with type 1 hernias and select patients with type 2 and smaller type 3 hernias underwent pH studies unless EGD demonstrated severe esophagitis or Barrett’s disease. If anything more than a 180-degree fundoplication was considered preoperatively, patients underwent formal esophageal manometry; after 2019, we began utilizing a less invasive screening marshmallow bagel esophagram to rule out dysmotility, with abnormal screening swallows requiring corroboration by follow-up invasive manometry. Preoperative risk assessment was determined using the American Society of Anesthesiologists (ASA) classification system. Clinical features including indication for surgery, surgical approach, and whether it was an elective, urgent or revision repair were also gathered through chart review. Patients with type I through type IV paraesophageal hernia who underwent repair with at least 6 months of follow-up were included in the study. Patients were divided into two comparison groups including those who underwent PEHR through conventional, standard repair versus patients who underwent PEHR with implementation of a critical view.

**Fig. 2:**
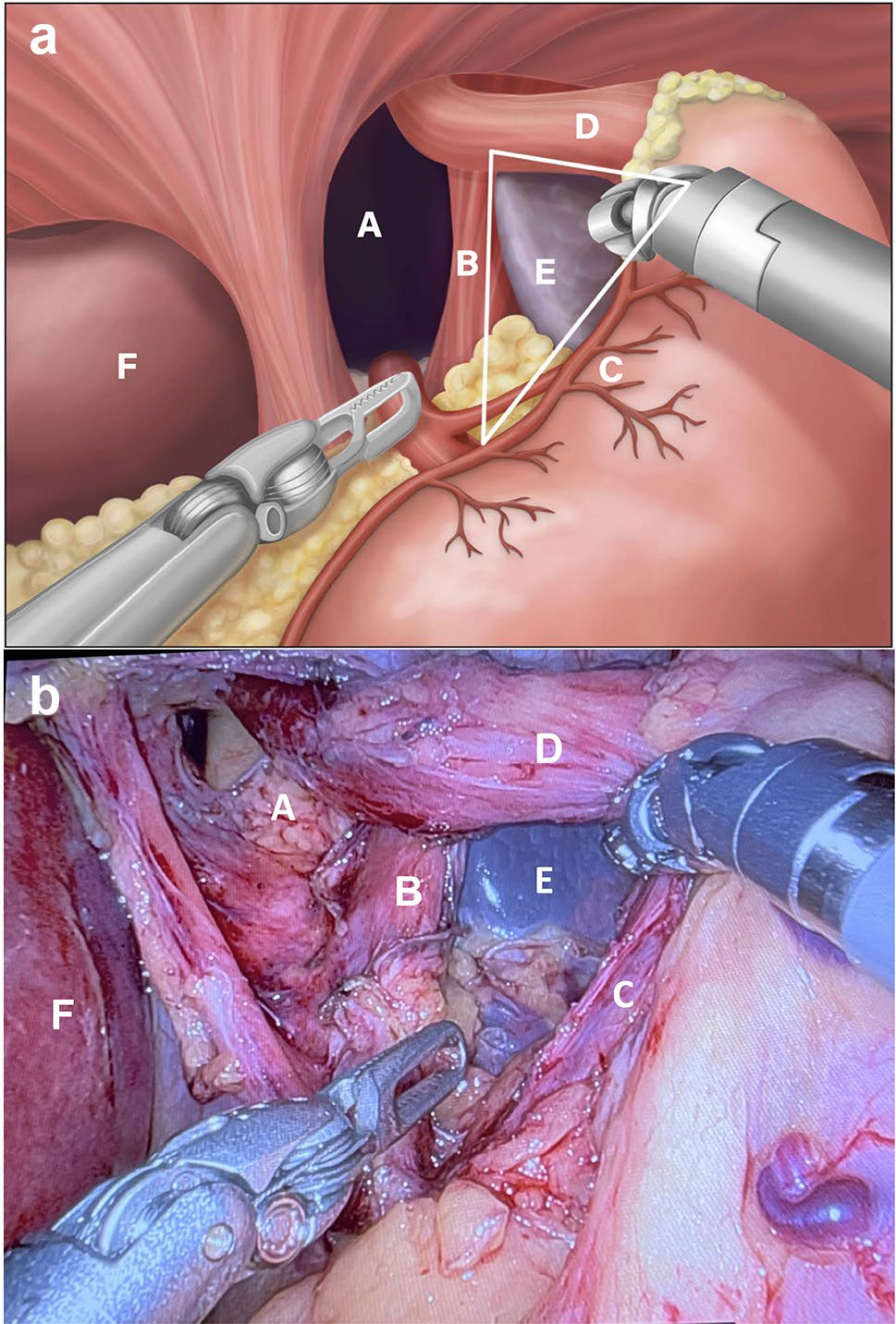
Critical view includes comprehensive dissection of (A) posterior mediastinum to reveal a dissected triangle whose borders include (B) left crus, (C) left gastric artery, and (D) distal esophagus. (E) Spleen should be visualized within the triangle and (F) liver is pictured for orientation. **a** Illustrated critical view of safety. **b** Intraoperative image of critical view in robotic paraesophageal hernia repair

Standard repair (SR) is defined as reduction of hernia contents to expose the hiatus, exposure of the right, and left crus of the diaphragm with dissection to separate the intraabdominal esophagus completely out of the mediastinum, posterior cruroplasty, and fundoplication. Critical view repair (CVR) was performed using similar steps as standard repair, but with emphasis on comprehensive dissection of the posterior mediastinum to expose up to the level of the inferior pulmonary veins, while completely separating all phrenoesophageal attachments posteriorly. The critical view is only achieved when a dissected triangle can be viewed whose borders include the left crus of the diaphragm, the left gastric artery, and the intrathoracic aorta with the spleen in the background (Fig. 2). This allows for adequate intraabdominal esophageal length and radicalization of the angle of His with a posterior curoplasty. A pseudo-gastric valve is then created through fundoplication with either a full wrap (Nissen) or a partial wrap depending on the patient’s preoperative studies.

Primary endpoints measured included hernia recurrence and reoperation rates. Secondary endpoints included symptom resolution, postoperative complications, intraoperative complications, and non-elective hospital readmission in a 30-day period. Operative time, discharge disposition, hospital length of stay, and outpatient follow-up duration were also analyzed to further understand outcomes within each cohort. Hernia recurrence was defined through radiographic studies. All patients in the critical view cohort received an upper gastrointestinal esophagram on postoperative day 1 as part of the aerodigestive program’s postoperative protocol. These patients were also provided follow-up at 1 week, 1 month, and 6 months postoperatively with their surgical team with further radiographic imaging ordered for patients with new or persistent symptoms.

All baseline characteristics were summarized with descriptive statistics. Quantile–quantile plots and Shapiro–Wilk testing were used to assess normality of variables. Student’s t tests and Wilcoxon rank-sum tests were performed to compare normally and non-normally distributed continuous variables, respectively, and Fisher exact tests or Pearson χ2 tests were used to compare categorical variables. Entropy balancing was performed to reduce intergroup differences in baseline covariates and thus control for potential confounders between SR and CVR patients with paraesophageal hernia (PEH) [18, 19]. Unadjusted bivariate analysis preceded entropy-balanced multivariate analysis. Selection of variables for incorporation into the entropy balancing algorithm was guided by Least Absolute Shrinkage and Selection Operator (LASSO) regularization [20]. Balance between the two patient groups pre- and post-entropy balancing was assessed by standardized mean differences. Following the application of entropy-balanced weights, multivariable logistic and linear regression models were developed to assess the independent association between CVR and outcomes of interest. Regression outputs were reported as adjusted odds ratios (AOR) or beta coefficients (β) with 95% confidence interval (CI). Additionally, an analysis of baseline characteristics associated with PEH recurrence by CVR status was performed via backward variable selection and multivariate regression. All variables that met threshold criteria (*P* = 0.200) were included for analysis in the final multivariate model. A *P* value < 0.05 was considered statistically significant. Statistical analyses were performed using Stata version 18.0 (StataCorp, College Station, Texas, USA).

## Results

A total of 297 patients who underwent PEHR at our institution between 2015 and 2023 met inclusion criteria. Among these patients, 207 (69.7%) utilized CVR whereas 90 (30.3%) underwent SR. Compared to SR, patients with CVR more commonly presented with Type I (36.9% CVR vs 18.9% SR, *P* < 0.001) or Type III (49.0% CVR vs 27.8% SR, *P* < 0.001) hernia. Average BMI was equivalent between groups, and comorbidities including diabetes, hypertension, coronary artery disease, and Barrett’s esophagus were also similar (Table 1). Gastro-esophageal reflux disease (GERD) resistant to medical therapy was the most common indication for PEHR in both groups (42.6% CVR vs. 43.2% SR). Overall, about 91% of patients underwent primary repair, with 9% of patients presenting for revision surgery (Table 1). Although not reaching statistical significance, there were more patients in the CVR cohort that presented for revision surgery (10.7% CVR vs. 4.4% SR, *P* = 0.081). There was a significant difference in operative approach between groups with robotic-assisted PEHR as the most common approach in patients who underwent CVR (81.1% CVR vs. 23.3%, *P* < 0.001). Patients with SR most commonly underwent laparoscopic repair, while the rate of open repair was comparable between both groups (Table 1). The most common fundoplication type in the overall cohort was anterior fundoplication which was more commonly used in CVR patients (67.8% CVR vs 27.8% SR, *P* < 0.001). All other baseline characteristics with univariate analysis are described in Table 1.

**Table 1:** Baseline characteristics of paraesophageal hernia repair

Characteristics	CVR (*n* = 207)	SR (*n* = 90)	Overall (*n* = 297)	*P* value
Age, median (IQR), years	66 (57–76)	65 (57–72)	66 (57–75)	0.358
BMI, mean (SD), kg/m^2^	30.0 (5.3)	29.9 (5.2)	30.0 (5.3)	0.829
Paraesophageal hernia type, *n* (%)				** < 0.001**
Type 1	76 (36.9)	17 (18.9)	93 (31.4)	
Type 2	3 (1.5)	3 (3.3)	6 (2.0)	
Type 3	101 (49.0)	25 (27.8)	126 (42.6)	
Type 4	19 (9.2)	13 (14.4)	32 (10.8)	
Unknown	7 (3.4)	32 (35.6)	39 (13.2)	
Indication for repair, *n* (%)				0.078
Dysphagia	56 (29.5)	28 (31.8)	84 (30.2)	
Regurgitation	13 (6.8)	8 (9.1)	21 (7.6)	
Dyspepsia	18 (9.5)	12 (13.6)	30 (10.8)	
Dyspnea	22 (11.6)	2 (2.3)	24 (8.6)	
GERD	81 (42.6)	38 (43.2)	119 (42.8)	
Repair approach, *n* (%)				
Open	30 (14.6)	14 (15.6)	44 (14.9)	0.826
Laparoscopic	9 (4.4)	55 (61.1)	64 (21.6)	** < .001**
Robotic	167 (81.1)	21 (23.3)	188 (63.5)	** < .001**
Repair type, *n* (%)				0.081
Primary	184 (89.3)	86 (95.6)	270 (91.2)	
Revision	22 (10.7)	4 (4.4)	26 (8.8)	
Wrap type, *n* (%)				** < 0.001**
Mesh	2 (1.0)	8 (8.9)	10 (3.4)	
Dor	139 (67.8)	25 (27.8)	164 (55.6)	
Nissen	24 (11.7)	19 (21.1)	43 (14.6)	
Toupet	1 (0.5)	10 (11.1)	11 (3.7)	
Belsey	12 (5.9)	2 (2.2)	14 (4.8)	
Unspecified	27 (13.2)	26 (28.9)	52 (18.0)	
Comorbid conditions, *n* (%)				
Diabetes	31 (15.1)	13 (14.4)	44 (14.9)	0.893
Hypertension	130 (63.1)	48 (53.3)	178 (60.1)	0.114
Coronary artery disease	29 (14.1)	15 (16.7)	44 (14.9)	0.596
GERD	130 (63.1)	58 (64.4)	188 (63.5)	0.826
Barrett’s esophagus	19 (9.2)	7 (7.8)	26 (8.8)	0.825
Atrial fibrillation	19 (9.2)	2 (2.2)	21 (7.1)	**0.046**
Other dysrhythmia	10 (4.9)	5 (5.6)	15 (5.1)	0.779
Proton pump inhibitor use	158 (76.7)	76 (84.4)	234 (79.1)	0.116
H2 blocker use	58 (28.2)	11 (12.2)	69 (23.3)	**0.003**
No GERD medication	36 (17.5)	12 (13.3)	48 (16.2)	0.493
History of thoracic surgery	18 (8.8)	5 (5.6)	23 (7.8)	0.480
History of abdominal surgery	110 (53.4)	52 (58.4)	162 (54.9)	0.426
Smoker	85 (41.3)	39 (43.3)	124 (41.9)	0.740

Bold indicates significance (*P* < 0.05)

*BMI* body mass index, *CVR* critical view repair, *SR* standard repair, *GERD* gastroesophageal reflux disease, *H2* histamine type 2, *IQR* interquartile range

Unadjusted perioperative outcomes between patients who underwent PEHR with CVR and SR are reported in Table 2. PEHR with CVR had a lower operative time (203 min CVR vs. 266 min SR, *P* < 0.001). Unadjusted rates of hernia recurrence were significantly lower in patients who underwent PEHR with CVR compared to SR (9.7% CVR vs. 22.2% SR, *P* = 0.005) and patients with CVR also experienced lower reoperation rates (0.5% CVR vs 10.0% SR *P* < 0.001). However, there was no significant difference between groups in 30-day complications (17.7% CVR vs. 23.9% SR, *P* = 0.226). The incidence of intraoperative complications was minimal and also comparable between groups (1.5% CVR vs. 2.3% SR, *P* = 0.633). Although there was no difference in unadjusted rates of symptom resolution between groups (87.8% CVR vs. 89.9% SR, *P* = 0.608), patients with CVR were found to have higher rates of antacid therapy cessation (34.6% CVR vs. 18.0%, *P* = 0.004). Unadjusted rates of non-elective readmission and non-home discharge were equivalent between groups and are described in Table 2. To evaluate experience bias in the CVR group, a subgroup analysis after the first 96 (of the 207) patients was performed and found that the recurrence rate was 11.6% at that time. With the experience of another 111 cases, this recurrence rate was reduced to 9.7%.

**Table 2:** Unadjusted outcomes of PEHR

Outcome	CVR (*n* = 207)	SR (*n* = 90)	*P* value
Operative time, median (IQR), minutes	203 (141–250)	266 (230–328)	** < 0.001**
Intraoperative complications, *n* (%)	3 (1.5)	2 (2.3)	0.633
GERD resolution, *n* (%)	180 (87.8)	80 (89.9)	0.608
Resolution of dysphagia, *n* (%)	193 (93.2)	82 (91.1)	0.881
30-day complications, *n* (%)	36 (17.7)	21 (23.9)	0.226
Length of stay, median (IQR), days	1 (0–3)	1 (1–4)	**0.019**
Reoperation, *n* (%)	1 (0.5)	9 (10.0)	** < 0.001**
Non-elective readmission, *n* (%)	26 (12.6)	10 (11.1)	0.715
Non-home discharge, *n* (%)	18 (8.7)	7 (7.8)	0.785
Hernia recurrence, *n* (%)	20 (9.7)	20 (22.2)	**0.005**
Cessation of GERD medication, *n* (%)	71 (34.6)	16 (18.0)	**0.004**

Bold indicates significance (*P* < 0.05)

*PEHR* paraesophageal hernia repair, *IQR* interquartile range, *CVR* critical view repair, *SR* standard repair, *GERD* gastroesophageal reflux disease

Multivariate adjustment using entropy balancing generated a strongly balanced distribution of baseline covariates between surgical treatment groups (standardized mean differences ranged from − 1.79 × 10^–14^ to 7.09 × 10^–15^). Following incorporation of entropy-balanced sample weights, patients who underwent PEHR with CVR were found to have an approximately tenfold reduction in hernia recurrence (AOR 0.08, 95% CI 0.03–0.23, *P* < 0.001). Risk of reoperation was similarly demonstrated to be reduced in the CVR cohort (AOR 0.04, 95% CI 0.01–0.08, *P* < 0.001). CVR was associated with an approximately 1-day reduction in length of stay (*β* = − 1.23, 95% CI − 2.44 to − 0.02, *P* = 0.046). Perioperative outcomes including 30-day postoperative complications and hospital readmission also had a lower likelihood in patients who underwent CVR (Table 3). Although unadjusted bivariate analysis revealed no difference in resolution of dysphagia between CVR and SR, on multivariate analysis CVR was associated with lower odds of dysphagia (AOR 0.26, 95% CI 0.08–0.82, *P* = 0.022). There was no association between CVR and resolution of GERD symptoms, cessation of antacid therapy, operative time, or intraoperative complications (Table 3).

**Table 3:** Adjusted outcomes of Critical View cohort for paraesophageal hernia repair

Outcome	AOR/β	95% CI	*P* value
Operative time, β, minutes	− 5.81	− 60.77 to 49.15	0.835
Intraoperative complications, AOR	0.13	0.02–1.08	0.059
Symptom resolution, AOR	0.31	0.07–1.44	0.134
Dysphagia, AOR	0.26	0.08–0.82	**0.022**
30-day complications, AOR	0.13	0.05–0.31	** < 0.001**
Length of stay, β, days	− 1.23	− 2.44 to − 0.02	**0.046**
Reoperation, AOR	0.04	0.01–0.08	** < 0.001**
Non-elective readmission, AOR	0.16	0.06–0.44	** < 0.001**
Non-home discharge, AOR	0.28	0.09–0.90	**0.033**
Hernia recurrence, AOR	0.08	0.03–0.23	** < 0.001**
Cessation of GERD medication, AOR	1.02	0.28–3.64	0.997

Bold indicates significance (*P* < 0.05)

*AOR* adjusted odds ratio, *GERD* gastroesophageal reflux disease, *CI* confidence interval

On multivariate analysis, comorbidities including BMI of 30–34.9 kg/m^2^ (HR = 5.63, 95% CI 1.51–8.98, *P* = 0.022), longstanding GERD (HR = 5.18, 95% CI 2.13–8.18, *P* = 0.007), and history of thoracic surgery (HR = 4.89, 95% CI 1.36–6.33, *P* = 0.027) were found to relate to increased risk of hernia recurrence. There was no association with hernia recurrence in patients with a BMI > 35 kg/m^2^, although these numbers of patients were relatively low (BMI 35–40 kg/m^2^ [*n* = 33, 11%] and BMI > 40 kg/m^2^ [*n* = 13, 4.3%]). Revision repair and surgical approach (open, laparoscopic, and robotic) did not demonstrate an association with hernia recurrence (Table 4). Toupet repairs were found to be associated with a decreased risk of hernia recurrence (AOR 0.23 95% CI 0.00–0.08, *P* < 0.001) but were used minimally in our cohort (*n* = 11, 3.7%).

**Table 4:** Multivariate analysis of risk of paraesophageal hernia recurrence by baseline covariates

Covariate	HR	95% CI	*P* value
Age			
< 45	REF	–	–
45–60	0.15	0.01–2.72	0.200
61–80	1.24	0.08–18.37	0.875
> 80	1.89	0.23–10.61	0.155
BMI			
< 30	REF	–	–
30–34.9	5.63	1.51–8.98	**0.022**
35–40	3.33	0.36–3.99	0.291
> 40	2.07	0.05–11.13	0.449
Paraesophageal hernia type			
Type 1	REF	–	–
Type 2	2.90	0.03–4.29	0.638
Type 3	0.07	0.01–0.40	**0.003**
Type 4	0.42	0.07–2.46	0.336
Repair type			
Primary	REF	–	–
Revision	0.16	0.02–1.76	0.135
Repair approach			
Open	REF	–	–
Laparoscopic	2.94	0.47–3.97	0.199
Robotic	0.86	0.08–4.87	0.902
Wrap type			
Dor	REF	–	–
Belsey	0.53	0.04–6.62	0.626
Mesh	1.81	0.00–6.99	0.853
Nissen	1.62	0.15–7.50	0.691
Toupet	0.23	0.00–0.08	** < 0.001**
Diabetes	3.44	0.62–8.94	0.156
Hypertension	1.26	0.22–6.07	0.796
Coronary artery disease	0.23	0.03–1.54	0.129
GERD	5.18	2.13–8.18	**0.007**
Barrett’s esophagus	0.87	0.11–6.92	0.892
Other dysrhythmia	0.03	0.00–2.33	0.113
Proton pump inhibitor use	0.86	0.17–4.41	0.853
H2 blocker use	2.37	0.32–7.84	0.401
Smoker	0.89	0.22–3.59	0.870
History of thoracic surgery	4.89	1.36–6.33	**0.027**
History of abdominal surgery	0.41	0.07–2.42	0.326

Bold indicates significance (*P* <0.05)

*BMI* body mass index, *CI* confidence interval, *CVR* critical view repair, *GERD* gastroesophageal reflux disease, *HR* hazard ratio, *H2* histamine type 2

## Discussion

**Fig. 3:**
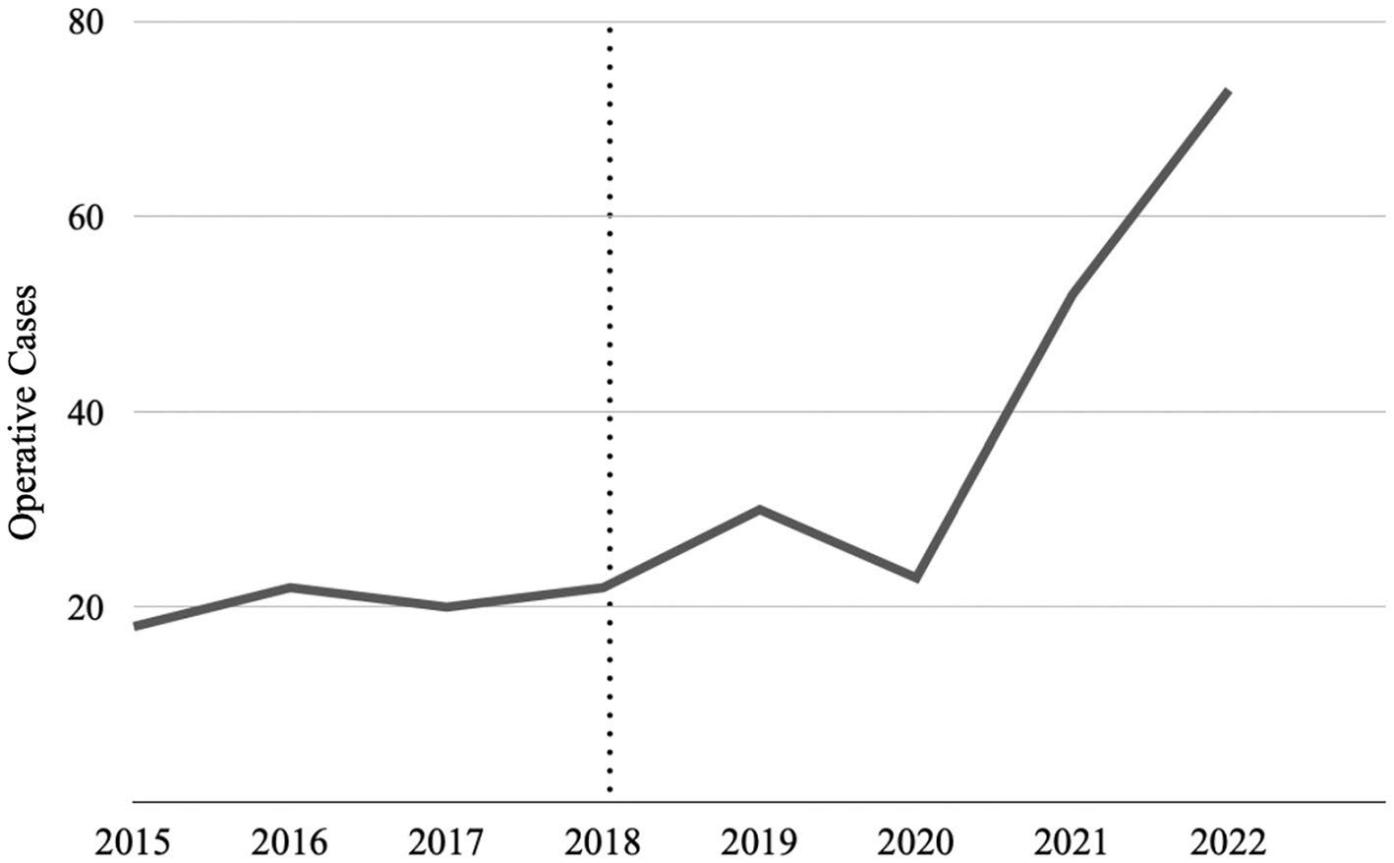
Operative volume following the development of an aerodigestive program

PEHR with CVR utilization revealed improved operative outcomes and was associated with lower rates of hernia recurrence and reoperation rates. Hernia recurrence was reduced to less than 10% in patients who underwent repair with CVR, which is lower than previously reported recurrence rates [5, 21]. PEHR with CVR led to decreased postoperative complications and less likelihood of hospital readmissions. Length of hospital stay was consistently reduced in the CVR cohort to 1 day postoperatively, which was partly due to the initiation of the aerodigestive program at our institution. The critical view was devised and implemented following the creation of our institution’s aerodigestive program to address complex foregut care and to help standardize the operation at our academic teaching hospital. Through the creation of a multidisciplinary aerodigestive program, patients presenting with a paraesophageal hernia underwent a standardized preoperative workup that allowed for more individualized repair. The aerodigestive program helps increase the operative cases (Fig. 3), which resulted in greater operative exposure and mastery of the critical view. Better outcomes drive more cases which in turn further improves outcomes.

Implementation of a critical view did not add additional operative time and is a learnable technical application that can be used in all hernia types irrespective of surgical approach. Direct comparisons of operative times in the early and later cohorts are challenging given experience bias on the part of the entire aerodigestive team and with improvements in robotic platforms through the years. As a minimally invasive program, we did not find statistical outcome differences between surgical approaches at our institution with respect to hernia recurrence. Laparoscopic repair has the benefits of minimally invasive surgery, including reduced postoperative pain, lower morbidity, and shorter hospital stays compared to open procedures [11]. However, laparoscopy presents recognized challenges, such as limited range of motion (especially for suturing and tough mediastinal dissection) for straight laparoscopic instruments, two-dimensional imaging, and suboptimal ergonomics for surgeons [7]. Robotic surgery addresses these challenges by providing improved three-dimensional optics, enhanced dexterity with endo-wrist movements, and improved ergonomics for surgeons. Robotic PEHR is the current standard of care at our institution due to surgeon preference, with laparoscopy and open repair reserved for specific cases. Other studies corroborate our findings and have also not found a difference in perioperative outcomes in laparoscopic versus robotic repair [22, 23].

Hernia recurrence can be classified as early (within the first 6 months of surgery) or late (occurring greater than 6 months). Early recurrences are often technical and can occur for multiple reasons including the wrap construction being too tight leading to dysphagia and need for dilation or re-repair, crural closure failure, inadequate intraabdominal esophagus, or incomplete removal of the gastroesophageal fat pad [24]. Through skeletonizing the structures seen in the critical view which includes visualization of posterior mediastinum to the level of the inferior pulmonary veins, the left crus of the diaphragm, the left gastric artery, and the spleen, adequate intraabdominal esophageal length can be achieved so that many of these technical errors can be avoided. The critical view also provides opportunity to anteriorly displace the angle of His with an aggressive posterior cruroplasty. By performing these steps, we can capitalize on a two-valve hypothesis to prevent GERD by encouraging crural apposition along the intraabdominal esophagus well proximal to an externally augmented lower esophageal sphincter by fundoplication. Radicalization of the angle of His further makes breakthrough GERD symptoms less common. With this learnable, reproducible technique, we have shown recurrence rate for all PEHR to be below 10%.

The incidence of patients requiring reoperative repair is much lower. Many patients that have a small radiographic recurrence of their PEHs have minimal symptoms and can be observed. For patients that have mild to moderate symptoms, many can be managed with a combination of antireflux therapy and diet modification. However, it is recommended that those with significant or persistent symptoms, or those that have a large recurrent type II or III PEH, or any recurrent type IV PEH, undergo repair [22]. In our study, recurrence was diagnosed through postoperative radiographic studies, including barium swallow studies and CT imaging. Indication for reoperation followed the protocol described and was implemented in both surgical groups. Revision repair is estimated to occur in approximately 15% of all paraesophageal hernia recurrences [24, 25]. Our study had a similar reoperation rate, with 10.2% of all recurrences in the SR cohort requiring a reoperation. The reoperation rate in the CVR group was incredibly low (0.5%) and may be an underestimate since follow-up may have been a limit in this study.

Resolution of symptoms were measured as resolution of dysphagia, resolution of reflux, and cessation of antacid therapy. Patients who underwent repair with CVR were found to have a significant reduction in dysphagia in comparison to patients who underwent SR; however, there was no difference in resolution of reflux in either patient group. Cessation of antacid therapy was higher in patients who underwent CVR repair, but these differences were not found to be significant on adjusted multivariate analysis likely due to high number of patient charts having no information on current status of antacid use.

Our study has some important limitations. Critical view repair is compared to historic control (standard repair) by way of a retrospective single institution cohort of patients. Prospectively collected data (preferably randomized) would have provided a more reliable and less biased head-to-head comparison. Unfortunately, considering our reported data with the current CVR, such a study is unlikely to gain IRB approval today. Secondly, the multidisciplinary aerodigestive team currently utilizes a limited number of specialized thoracic and general minimally invasive surgeons, which helps reduce variability in outcomes. This was not the case early on before 2018, as there were more surgeons performing this operation. As expertise among this group grew between 2015 until 2023, there may be other technical and experience-related factors that may have improved surgical outcome for this heterogeneous disease aside from the critical view. Thirdly, we certainly concede that there is considerable redistribution of proportion of type 3 paraesophageal hernias in the CVR group compared to type 1 hernias in the SR group; these were performed mostly by thoracic surgeons who were hired later into the aerodigestive program to compliment an already experienced group of minimally invasive foregut surgeons. In 2018, when we created a formal aerodigestive program, we agreed to utilize SAGES guidelines for classifying hernia type (1–4) clearly in the operative dictation so that we can better keep track of hernia type-specific outcomes and fuel future studies, avoiding “hernia unknown” mischaracterization and decrease comparative bias. Finally, determination of recurrence of symptoms through retrospective chart review is not entirely accurate. For example, cessation of antacid therapy is often at the discretion of the patient’s gastroenterologist and may have occurred at a later time in the outpatient setting that may not have been captured in the chart review or conversely may not have been reported at all. GERD quality of life questionnaires have been inconsistently used in years past at our institution and may be useful for future studies. Perhaps these surveys coupled to imaging at 6 and 12 months postoperatively in a prospective design could provide an interesting comparison of symptom to imaging recurrence, which do not always mirror one another in real life.

We present a novel critical view technique that results in favorable postoperative outcomes including a reduction in recurrence and reoperation in paraesophageal hernia repairs. This technique is teachable to trainees and reproducible across multiple surgical specialties and approaches. It does not add time to the operative case and is associated with improvement in length of stay and reoperation. Standardizing a sometimes difficult paraesophageal hernia repair embattled by significant recurrence rate is always a challenge, but broad implementation of the critical view may help improve outcomes for these oftentimes complicated patients.
